# Development of a complex amino acid supplement, Fatigue Reviva™, for oral ingestion: initial evaluations of product concept and impact on symptoms of sub-health in a group of males

**DOI:** 10.1186/1475-2891-12-115

**Published:** 2013-08-08

**Authors:** R Hugh Dunstan, Diane L Sparkes, Tim K Roberts, Marcus J Crompton, Johan Gottfries, Benjamin J Dascombe

**Affiliations:** 1 School of Environmental and Life Sciences, University of Newcastle, Callaghan, NSW 2308, Australia; 2 The Tom Farrell Institute for the Environment, University of Newcastle, Callaghan, NSW 2308, Australia; 3 Department of Chemistry and Molecular Biology, Gothenburg University, Gothenburg, Sweden; 4 School of Environmental and Life Sciences, University of Newcastle, Ourimbah, NSW 2258, Australia

**Keywords:** Sub-health, Fatigue, Amino acid, Dietary supplement

## Abstract

**Background:**

A new dietary supplement, Fatigue Reviva™, has been recently developed to address issues related to amino acid depletion following illness or in conditions of sub-health where altered amino acid homeostasis has been associated with fatigue. Complex formulations of amino acids present significant challenges due to solubility and taste constraints. This initial study sets out to provide an initial appraisal of product palatability and to gather pilot evidence for efficacy.

**Methods:**

Males reporting symptoms of sub-health were recruited on the basis of being free from any significant medical or psychological condition. Each participant took an amino acid based dietary supplement (Fatigue Reviva™) daily for 30 days. Comparisons were then made between pre- and post-supplement general health symptoms and urinary amino acid profiles.

**Results:**

Seventeen men took part in the study. Following amino acid supplementation the total Chalder fatigue score improved significantly (mean ± SEM, 12.5 ± 0.9 versus 10.0 ± 1.0, *P*<0.03). When asked whether they thought that the supplement had improved their health, 65% of participants responded positively. A subgroup of participants reported gastrointestinal symptoms which were attributed to the supplement and which were believed to result from the component fructooligosaccharide. Analysis of urinary amino acids revealed significant alterations in the relative abundances of a number of amino acids after supplementation including an increase in valine, isoleucine and glutamic acid and reduced levels of glutamine and ornithine. Discriminant function analysis of the urinary amino acid data revealed significant differences between the pre- and post-supplement urine excretion profiles.

**Conclusions:**

The results indicated that Fatigue Reviva™ was palatable and that 65% of the study group reported that they felt the product had improved their health. The product could provide an effective tool for the management of unexplained fatigue and symptoms of sub-health. Further product development may yield additional options for those patients susceptible to fructooligosaccharide.

## Background

The World Health Organisation defines health as “a state of complete physical, mental and social well-being and not merely the absence of disease or infirmity” [1]. In contrast, sub-health has been described as a chronic condition of unexplained deteriorated physiological function which falls between health and illness [2]. Sub-health is usually indicated by the presence of lowered energy levels, loss of vitality, altered sleeping patterns and an increased incidence of viral infections all occurring in the absence of a defined disease diagnosis [2]. The more severe cases of sub-health may present as conditions such as chronic fatigue syndrome (CFS) and fibromyalgia. In the past, interest in the treatment of fatigue has focussed on the more severe fatigue states of cancer-related fatigue (CRF) and CFS. The aetiology of both CFS and CRF remain largely unknown and no effective treatment strategies have yet been agreed upon. There is an increasing need for scientifically validated therapies which can effectively and simply treat the symptoms of sub-heath including fatigue.

Research into the underlying causes of CFS and CRF has revealed the presence of altered amino acid homeostasis in these conditions [3-6]. Altered amino acid levels have been demonstrated by the current research team in association with sub-health conditions including chronic fatigue [3] and fatigue in radiotherapy breast cancer patients [6]. In a relatively large study, depletion of excreted amino acids in CFS was demonstrated in comparison to non-fatigued controls [7]. Results of metabolic profiling have pointed to the presence of a chronic catabolic state associated with sub-health. A catabolic state is usually acute and transient and may occur in response to trauma, physical exertion or infection. It has been proposed that in sub-health, a long-term catabolic state exists resulting in cellular malnutrition as evidenced by particular deficits in amino acids [2]. Dunstan [8] demonstrated that a range of different states of phenotypic amino acid homeostasis exist, where some may require higher dietary levels of particular amino acids. An increased requirement for amino acids may not be limited to the essential amino acids. Although essential amino acids must be derived from the diet as they cannot be synthesised by humans, some non-essential amino acids may also become “conditionally essential” as the body’s rate of synthesis may not meet the required demand. If these nutritional demands are not met, increased susceptibility to environmental challenges may occur and a state of sub-health may result [8].

Amino acid supplementation has been shown to stimulate the immune system [9,10], enhance recovery of the critically ill [11] and maintain gut function [12] as well as alleviate symptoms of CFS [5] and CRF [13]. During exercise, supplementation with branched chain amino acids (BCAAs) has been shown to improve performance, possibly through a reduction in fatigue [14]. It has been proposed that as a result of supplementation, changes in the plasma tryptophan to BCAA ratio could affect CNS uptake of tryptophan and resultant 5-hydroxytryptamine synthesis which is believed to be involved in fatigue during prolonged exercise [14]. Supplementation of mice with BCAAs has also been shown to improve physical endurance and motor coordination and increased life span with an associated increase in cardiac and skeletal muscle mitochondrial biogenesis and function [15]. Branched chain amino acid levels have been shown to be reduced in CFS with deficits in BCAA availability having the capacity to impact upon brain function via CNS glutamate and glutamine homeostasis [7].

A dietary supplement, Fatigue Reviva™, has recently been developed by TOP Nutrition Pty Ltd to address issues related to amino acid depletion in conditions of sub-health. Development of a product endeavouring to provide broad-spectrum amino acid supplementation with the associated vitamins and cofactors required for effective utilisation, presents significant challenges. The supplement mixture needs to be formulated to comply with food industry standards, accommodate solubility and palatability issues, and have minimal adverse impacts. This initial study represents a preliminary phase of testing of Fatigue Reviva™ to appraise palatability and feedback on the use of the product whilst gathering pilot evidence on the potential to reduce symptoms of fatigue. If further product development was required as a result, this should be completed before a larger scale double-blinded and placebo-controlled trial would be implemented. This first stage of product development investigated the use of Fatigue Reviva™ over a thirty-day period in a group of healthy men experiencing some symptoms of sub-health. Symptoms were appraised and urine samples were analysed for amino acid composition before and after the supplementation period. Feedback on palatability and usage were recorded and collated at the end of the supplementation period for each participant.

Since the product under development was not a drug, this investigation did not fall under the phase 1 or phase 2 definitions of a drug trial. Safe daily doses of each of the amino acids have already been determined and dosage limits are governed by the NSW Food Authority restrictions. The product under testing was a food category product. All ingredients used in this product conform to the regulations set out in the Foods Standards Code (FSANZ) [16], the NSW Food Act 2003 [17] and the NSW Food Registration 2010 [18]. To reduce heterogeneity within this initial group, only male participants were included. The study will be further extended in the future to include females.

## Methods

Sub-health was defined as subjects experiencing reduced energy levels, change in sleeping patterns, increased incidence of infections and loss of vitality. Subjects who reported experiencing symptoms of sub-health but were otherwise healthy were recruited from patients of health practitioners and from the general public. The majority of participants (n=15) were recruited from the general public. Potential participants were excluded from the study if they reported a previous or current diagnosis of a significant medical (e.g. heart disease) or psychiatric (e.g. depression) condition. Two participants were excluded on the basis of mental illness. Participants were required to take 20g of the amino acid based dietary supplement (Fatigue Reviva™) blended in 100 ml of water each day for 30 days. The nutritional supplement comprised an amino acid complex, containing both essential and non-essential amino acids at doses compliant with the Australian Food Standards Code, the NSW Food Act 2003 and the NSW Food Registration 2010, carbohydrates including fructooligosaccharide (FOS) and vitamins and minerals. Prior to supplementation and the day following the completion of supplementation, subjects were required to provide a fasted first-morning urine sample collected in a Urine-Monovette® 10 ml boric acid tube (Sarstedt, Germany) and to complete two questionnaires. Participants were assessed for fatigue using the Chalder fatigue scale [19] (11-items, Likert scored). Participants were also assessed for current general health and well-being using an 86 item questionnaire developed and extensively used by the current research team [20-22]. The questionnaire assessed self-reported symptoms including fatigue, pain, gastrointestinal, cognitive, neurological and infection related symptoms. Participants were asked to indicate how much they had been affected by each of the symptoms over the last seven days. The questionnaire was Likert scored with responses ranging from 0 “not at all” to 4 “extremely”. Indices were then generated from the individual questions. The urinary amino acid analyses were performed using the commercial EZ:Faast™ derivatisation method (esterification of amino acids) followed by gas chromatography/flame ionisation detection (GC/FID) [6].

The sample size was based upon the research team’s experience of amino acid profiling and the effects of supplementation in humans and was projected to provide sufficient power to determine whether the supplement had the potential to alter amino acid homeostasis. Comparisons were made between the pre- and post-supplement symptom and amino acid profiles of the cohort. The preliminary nature of the study meant that a placebo group was not included at this stage of product assessment. Statistical analyses were performed using Statistica™ release 7.0 (Statsoft Inc., Tulsa, USA). NVivo 9 (QSR International Pty Ltd.) for qualitative data was used to assess open-ended questions regarding supplement experience. Mann–Whitney U test was used to analyse the Chalder fatigue scale scores. Amino acid data were assessed using forward stepwise discriminant function analysis and Mann–Whitney U test performed on arcsine transformed data. Levels of statistical significance were set at *P*<0.05.

The study was approved by the University of Newcastle Human Research Ethics Committee (H-2010-1313) and was registered with the Australian New Zealand Clinical Trials Registry (ACTRN12611000403932). All participants provided informed written consent before taking part in the trial.

## Results and discussion

Seventeen men reporting symptoms of sub-health were enrolled in the current study. Potential participants were included in the study if they were reportedly free of a diagnosis of any significant medical condition. Amongst the men included in the study, one participant reported controlled hypertension and another reported having previously suffered with sleep apnoea/narcolepsy but indicated that they were currently free from any significant medical condition. The mean age of the subjects was 37.6 ± 11.7 years (mean ± SD, range 19–65 years) and the mean body mass index was 27.7 ± 4.2 kg/m^2^.

Following 30 days of amino acid supplementation, a significant reduction was seen in the evaluations of total fatigue and physical fatigue scores compared with the initial evaluations prior to supplementation (Table 1). These reductions in scores from the Chalder fatigue questionnaire represented reduced levels of fatigue in subjects following supplementation with Fatigue Reviva™ in this group of relatively healthy men reporting symptoms of sub-health.

**Table 1:** Chalder fatigue scale scores (mean ± SEM) for a group of 17 males before and after supplementation with amino acids

**Chalder fatigue scale**	**Before supplement**	**After supplement (30 days)**	***P *value**
	Mean (SEM)	Mean (SEM)	
**Total fatigue**	**12.5 (0.9)**	**10.0 (1.0)**	***P *< 0.03**
**Physical fatigue**	**7.6 (0.5)**	**6.1 (0.7)**	***P *< 0.03**
Mental fatigue	4.8 (0.5)	3.9 (0.3)	*ns*

Statistical test: Mann–Whitney U test, *P* < 0.05.

The participants were asked to provide answers to questions regarding their experience of using the supplement in an endeavour to determine whether the product required further development. When asked “If given the opportunity would you continue to use the supplement?” 12 of 16 men (75%) indicated that they would continue. In addition, when asked “Do you think that the dietary supplement improved your health?” 11 of the 17 men (65%) responded “Yes” whilst six indicated “No”. Participants were also invited to provide comments regarding the ability of the Fatigue Reviva™ supplement to improve their health (Table 2). Five of the participants provided comments indicating that the supplement had increased their levels of energy or reduced tiredness (Table 2).

**Table 2:** Types and number of comments regarding perceived improvement in health provided by participants after 30 days of dietary supplementation

**Comments**	**Number of participants**
**Positive response**	
Increased energy	4/17
Reduced fatigue	1/17
More alert	1/17
Aided recovery from mild illness	1/17
Reduced muscle soreness	1/17
Initial improvement	1/17
Minor change	1/17
**Negative response**	
No change	4/17
GIT symptoms (flatulence)	1/17
Fatigue not improved	1/17

Note: Thirteen participants provided a comment/s.

When participants were asked to “Please describe your experience of using the supplement” 5 of the subjects provided positive comments indicating that they felt the supplement had improved their levels of energy (Table 3) whilst one of the men specifically reported an improvement in gastrointestinal tract (GIT) symptoms. Amongst the negative responses provided, 4 participants indicated that they had experienced GIT symptoms when using the supplement. These four individuals attributed an increase in abdominal pain/discomfort, flatulence and in one instance diarrhoea to supplement use (Table 3).

**Table 3:** Types and number of comments regarding experience of supplement use provided by participants after 30 days of dietary supplementation

**Comments**	**Number of participants**
**Positive response**	
Energy increase	5/17
Exercise recovery	1/17
GIT improvement	1/17
Initial improvement	1/17
More alert	1/17
Overall good	4/17
Taste	3/17
**Negative response**	
GIT symptoms*	4/17
No benefits	2/17
Solubility	2/17
Taste	1/17
Weight gain	1/17
**GIT symptom - negative**	
Diarrhoea	1/17
Flatulence	2/17
Pain/discomfort	2/17

Note: Fifteen participants provided a comment/s. *“GIT symptoms” was further broken down into the types of symptoms reported.

Changes in GIT symptoms, both negative and positive, may be attributed to the composition of the supplement. In addition to amino acids, the nutritional supplement contained the prebiotic fructooligosaccharide (FOS) [23]. The ingestion of FOS has been shown to promote the growth of gastrointestinal bifidobacteria which are thought to have a number of health benefits including lowering blood levels of neurotoxic ammonia [24]. The FOS was considered an important component as it is fermented by the gut bacteria in the large intestine resulting in the formation of short-chain fatty acids, which can also have beneficial effects. These short chain fatty acids can potentially stimulate the growth of bacteria, including lactobacilli and bifidobacteria [25]. The FOS also played an important role in maintaining the palatability of the supplement without direct uptake by the human body and thus provided vital functions of sweetener and GIT bacterial enhancement, without significant impact on sugar loading and avoiding the use of artificial sweeteners.

Five (5) out of 17 subjects reported some adverse symptoms including flatulence and discomfort, which has been previously reported with FOS use at much higher doses [26]. In contrast, other subjects in this study reported benefits including reduced bowel irritation and GIT improvement which may be associated with the potential enhancement of bifidobacteria which have been reported to be mediated by FOS [23]. The symptom questionnaires and urine excretion data for these five subjects were further evaluated in comparison to the remaining 12 subjects to see whether any issues of sub-health might be identified as markers of individuals likely to experience GIT discomfort on supplementation. The 86-item symptom questionnaire enabled the evaluation of symptom groups or indices including a broadly based “neurological” index. This index included assessments of the following symptoms reported in the prior 7 days: faintness or dizziness; crying easily; tinnitus; photophobia; mind going blank; trouble concentrating; hypersensitivity; mental fatigue; and panic attacks. The mean neurological index score for the 5 subjects reporting the adverse GIT symptoms, was significantly higher prior to supplementation compared with the remaining 12 subjects as shown in Table 4 (*P*<0.004). It should be noted that the maximum score possible for the index was 10 which shows that the mean scores <3 by these subjects were consistent with the classification of experiencing only sub-health issues. Both GIT symptom subgroups recorded lower mean total fatigue scores following supplementation although only the GIT symptom free group recorded a statistically significant improvement in fatigue (Mann–Whitney U test, *P* < 0.04). Both pre- and post-supplementation, the sub-group reporting GIT symptoms recorded higher mean total fatigue scores than the GIT symptom free group. Although these findings did not reach levels of statistical significance, it is possible that with a larger cohort it may be shown that those individuals suffering from GIT symptoms also experience higher levels of fatigue. The group of 5 subjects with GIT symptoms was also characterised by increases in the relative abundances of asparagine, hydroxylysine and hydroxyproline in the urine excretion profile. Following supplementation these changes in amino acids, along with the neurological difficulties indicated by the increased neurological index score, were consistent with a process of normalisation for the 5 subjects reporting the GIT symptoms (Table 4).

**Table 4:** Differences between subjects reporting GIT problems and those who did not report GIT symptoms following supplementation

	**Pre-supplementation**			**Post-supplementation**		
**Measure**	**Subjects reporting GIT symptoms post-supplement (mean)**	**Subjects GIT symptom free post-supplement (mean)**	***P *value**	**Subjects reporting GIT symptoms post- supplement (mean)**	**Subjects GIT symptom free post-supplement (mean)**	***P *value**
**General health indices**						
Neurological index	1.3	0.5	< 0.004	1.0	0.4	*ns*
**Chalder fatigue scale**						
Total fatigue scores	14.2	11.8	*ns*	12.6	8.9	*ns*
**Amino acids**						
(Relative % abundance)						
Asparagine	6.60%	4.78%	< 0.03	6.11%	4.89%	*ns*
Hydroxylysine	1.62%	1.15%	< 0.05	1.72%	1.32%	*ns*
Hydroxyproline	0.58%	0.34%	< 0.04	0.65%	0.52%	*ns*

Statistical test: Mann–Whitney U. n = 5 and 12 for the participants reporting GIT symptoms and those who did not report GIT symptoms respectively. *ns* = statistically non-significant.

Future investigations should continue to determine whether these symptom profiles or urine excretion anomalies of GIT-sensitive subjects could be used as predictors for those people more likely to experience GIT issues with taking the Fatigue Reviva™ supplement. These predictors, if validated, may have future use to identify people with a lower tolerance for FOS and the development of a product with reduced FOS content may be warranted.

The analyses of the urine excretion profiles of amino acids pre- and post-supplementation for the entire cohort revealed significant increases in the relative abundances of isoleucine, valine, glutamic acid and proline following supplementation whilst significant reductions were seen in glutamine and ornithine (Table 5). Significant increases in the percentage of total essential amino acids and total BCAAs, which make up approximately one third of the essential amino acids in muscle proteins [27], were also seen after supplementation. These results were interpreted to reflect an altered homeostasis for amino acids in the body since the relative abundances in composition of urine were altered. The alterations in urinary excretion profiles did not reflect general increases in urinary amino acid output following elevated intake of the Fatigue Reviva™ supplement. This interpretation was supported by the significant decrease in glutamine observed after the supplementation period. Glutamine is a major component of the amino acid supplement and the diminished output relative to other amino acids supports the hypothesis of an altered homeostasis. Glutamine promotes the functioning of the immune system and is the most abundant free amino acid in plasma and muscle [28]. During stress there is an increase in glutamine efflux from skeletal muscle and it has been proposed that in catabolic states glutamine may become conditionally essential [29]. Provision of the broad-spectrum amino acid Fatigue Reviva™ supplement which includes glutamine, would have a potential impact to minimise proteolysis, support anabolism, promote function and growth of immune cells and alter the metabolic equilibrium in the body.

**Table 5:** Univariate analysis of the relative abundance of urinary amino acids and derivatives: comparison between participants pre- and post-supplementation

**Amino acid**	**Pre-supplement relative abundance % mean (SEM)**	**Post-supplement relative abundance % mean (SEM)**	***P *value**
Alanine	5.80 (0.34)	5.09 (0.32)	*ns*
Glycine	20.82 (1.08)	18.08 (1.58)	*ns*
α-aminobutyric acid	0.20 (0.05)	0.21 (0.04)	*ns*
**Valine**	**1.00 (0.06)**	**1.24 (0.08)**	**< 0.04**
β-aminoisobutyric acid	3.11 (0.49)	4.48 (1.14)	*ns*
Leucine	0.70 (0.04)	0.70 (0.04)	*ns*
**Isoleucine**	**0.17 (0.04)**	**0.35 (0.03)**	**< 0.02**
Threonine	2.45 (0.14)	2.35 (0.16)	*ns*
Serine	5.94 (0.39)	5.42 (0.37)	*ns*
**Proline**	**0.31 (0.07)**	**0.51 (0.08)**	**< 0.04**
Asparagine	5.32 (0.35)	5.25 (0.52)	*ns*
Aspartic acid	0.34 (0.08)	0.48 (0.06)	*ns*
Methionine	0.29 (0.04)	0.35 (0.04)	*ns*
Hydroxyproline	0.41 (0.07)	0.56 (0.08)	*ns*
**Glutamic acid**	**0.33 (0.07)**	**0.70 (0.09)**	**< 0.008**
Phenylalanine	1.07 (0.07)	1.20 (0.10)	*ns*
α-aminoadipic acid	1.86 (0.33)	1.65 (0.29)	*ns*
α-aminopimelic acid	0.15 (0.05)	0.10 (0.03)	*ns*
**Glutamine**	**12.61 (0.85)**	**8.75 (0.62)**	**< 0.003**
**Ornithine**	**1.22 (0.11)**	**0.99 (0.10)**	**< 0.04**
Glycine-proline dipeptide	2.06 (0.22)	1.99 (0.22)	*ns*
Lysine	6.53 (1.54)	6.36 (1.88)	*ns*
Histidine	17.70 (1.12)	22.59 (2.71)	*ns*
Hydroxylysine	1.29 (0.12)	1.44 (0.16)	*ns*
Tyrosine	1.92 (0.13)	2.07 (0.16)	*ns*
Proline-hydroxyproline dipeptide	3.04 (0.38)	2.84 (0.31)	*ns*
Tryptophan	1.31 (0.10)	1.40 (0.11)	*ns*
Cystathionine	0.86 (0.18)	1.37 (0.33)	*ns*
Cystine	1.21 (0.12)	1.47 (0.20)	*ns*
**Total EAA**	**31.22 (1.38)**	**36.55 (2.79)**	**< 0.05**
**Total BCAA**	**1.87 (0.13)**	**2.29 (0.11)**	**< 0.02**

Statistical test: Mann–Whitney U test, *P* < 0.05. Values are the mean (SEM) and are expressed as amino acid relative abundance (percentage). Analysis performed on arcsine transformed data. *ns* = statistically non-significant.

The alterations in the relative abundance composition of key amino acids supported a re-adjustment of amino acid homeostasis as opposed to a general increased throughput of ingested amino acids. The results of univariate analyses were supported by multivariate analysis of the urinary amino acid data. Discriminant function analysis of the urine excretion data revealed that it was possible to differentiate between the group’s pre- and post-supplement urine excretion profiles (Wilks’ Lambda = 0.07, *P*<0.0001). An ability to discriminate between groups based upon their pre- and post-supplement profiles may lead to a capacity in the future to apply urine testing to specifically identify those subjects who might benefit from supplementation with Fatigue Reviva™.

## Conclusion

The evaluation of the results for the cohort of 17 males indicated that the use of the Fatigue Reviva™ supplement for 30 days resulted in significantly reduced levels of fatigue and altered the corresponding urine excretion profiles. In total, 65% of the group reported that they perceived that the supplementation resulted in an improvement of their health. Only one subject reported substantial GIT symptoms, but a further 24% of the group reported some mild GIT symptoms of discomfort or bloating which was thought to be associated with the inclusion of the FOS. This group was also characterised prior to supplementation by significant elevations of symptoms such as “mind going blank”, and had elevated relative excretions of asparagine, hydroxylysine and hydroxyproline compared to the rest of the group. Conversely, other participants reported improvements in bowel irritation and GIT performance. An alternative supplement formulation with reduced FOS content was proposed, and the associated symptoms and urine excretion anomalies represent potential predictors for those likely to be GIT-sensitive to the supplement. The results of this pilot study indicated that the Fatigue Reviva™ has the potential to provide an effective tool for the management of unexplained fatigue and symptoms of sub-health.

## Abbreviations

CFS: Chronic fatigue syndrome; CRF: Cancer related fatigue; BCAA: Branched chain amino acid; GC/FID: Gas chromatography/flame ionisation detection; GIT: Gastrointestinal tract; FOS: Fructooligosaccharide; EAA: Essential amino acid.

## Competing interests

Hugh Dunstan and Tim Roberts work as consultants for the company Top Nutrition Pty Ltd to oversee a program of development for the amino acid supplement Fatigue Reviva™ trialled in the current study. Diane Sparkes has previously worked as a data-processing consultant for Top Nutrition Pty Ltd.

## Authors’ contributions

RHD and TR conceived the study, were responsible for the study design and the formulation of the dietary supplement. DLS contributed to the study design and performed the statistical analysis. RHD and DLS drafted the manuscript. MC carried out the urinary analysis and was involved in coordinating the study. BD was involved in coordinating the study and JG gave advice on data analysis. All authors were involved in interpretation of data, developing the content of the manuscript and revising multiple drafts prior to approval of the final manuscript.
